# Hepatitis B and C in dialysis units in Kosova

**DOI:** 10.1186/1743-422X-6-72

**Published:** 2009-06-04

**Authors:** Skender Telaku, Hajrullah Fejza, Ymer Elezi, Teuta Bicaj

**Affiliations:** 1 Gastroenterology Unit, University Clinical Center of Kosova, Prishtina, Republic of Kosova; 2 Sector for Public Health, Municipality of Prishtina, Republic of Kosova; 3 Nephrology Unit, University Clinical Center of Kosova, Prishtina, Republic of Kosova; 4 Department for Prosthetics Dentistry, University Dentistry Clinical Center of Kosova, Prishtina, Republic of Kosova

## Abstract

**Background:**

Hepatitis B virus (HBV) and hepatitis C virus (HCV) infections are important causes of morbidity and mortality of hemodialysis (HD) patients. This study aimed to investigate the epidemiological and clinical features of HCV and HBV infections in six different HD units in Kosova.

Five hundred and eighty-three end-stage kidney disease (ESKD) patients on maintenance HD from six HD centers in Kosova (358 female, 225 male, mean age 54,8 years (16–66) were included in this study. Data from databank of the National Blood Bank in Prishtina, as well as the data from the databank of the Transfusion Centers in Regional hospitals in Prizren, Peja, Gjilan, Mitrovica and Gjakova were taken in this study. Clinical data such as age, sex, HBsAg and anti-HCV antibody and primary causes of ESKD were examined.

Serological markers for HBV and HCV were determined with immunoenzymatic assay (ELISA).

**Results:**

The T-test and x^2 ^test were used to analyze the significance of the results. Among our HD patients HBsAg and anti-HCV antibody prevalence rate was 12%, respectively 43%. Chronic nephritis was a more frequent cause of ESKD among our HD patients. With unknown etiology were 23, 5% from them.

**Conclusion:**

HBV and HCV prevalence in our HD patients is still high. These data emphasize the need for stricter adherence to infection control, barrier precaution and preventive behaviors with all patients.

## Background

Hemodialysis (HD) patients are at high risk for viral hepatitis infections due to the high number of blood transfusion sessions, prolonged vascular access and the potential for exposure to infected patients and contaminated equipment [1,2].

The prevalence of HCV antibodies in nephrology units is high and has been reported to range from 5 to 54% [3]. HBV infection is less prevalent than HCV in HD units [4]. The rate of serum HBsAg seropositivity on maintenance HD in the developed world is currently low (0–10%) but outbreaks of acute HBV infection continue to occur in this setting. The prevalence of HBV infection within dialysis units in developing countries appears higher (2–20%) based on relatively several reports [5].

The aim of the present study was to investigate the epidemiological and clinical features of HCV and HBV infections in six different HD units in Kosova.

## Methods

Clinical and epidemiological data were obtained from January to December 2008 in six different HD units in Kosova (Prishtina with 150, Prizren with 110, Peja with 74, Gjilani 80, Mitrovica 71 and Gjakova with 31 patients). Data from databank of the National Blood Bank in Prishtina as well the data from the above mentioned transfusion centers were used in this study. Clinical data such as age, sex, HBsAg and anti-HCV antibody and primary cause of ESKD were examined.

Serological testing for HBV surface antigen and antibodies to HCV was performed using microparticle enzyme immunoassay (Abbott AxSM System, Abbott Laboratories, Abbott Park, Illinois, USA) and appropriate assays manufactured for the system (Abbott-AxSYM HBsAg version 2 and HCV version 3.0 assays). The T-test and x^2 ^test were used to analyze the significance of the results.

## Results

From 583 patients, 358 (61,4%) of them were female and 225(38,6%) were male, and the age ranged from 16–66 years with mean age 54.8 years, (Table 1).

**Table 1:** The number and sex of tested patients in Hemodialysis Unit

**Sex**	**N**	**%**
M	225	38.6

F	358	61.4

Total	583	100

**Figure 1:**
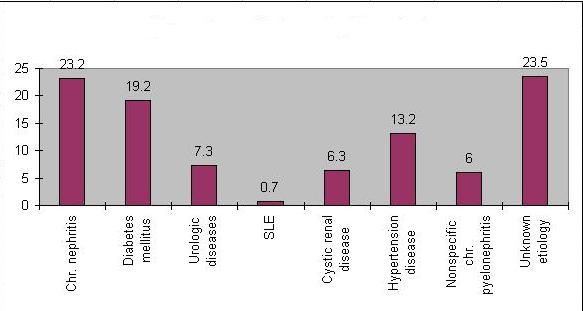
**Etiology of prevalent regular HD patients**.

The major primary renal diseases in the end stage of kidney disease (ESKD) patients included chronic nephritis (23, 2%), diabetes mellitus (19,2%), hypertension (13,2%), urologic diseases (7,3%), cystic renal diseases (6,1%), and nonspecific chronic pyelonephritis (6%). Four patients (0, 7%)were with systemic lupus erytemathosus (SLE). There were 23, 5% (137 patients) with unknown etiology, (Figure 1).

**Figure 2:**
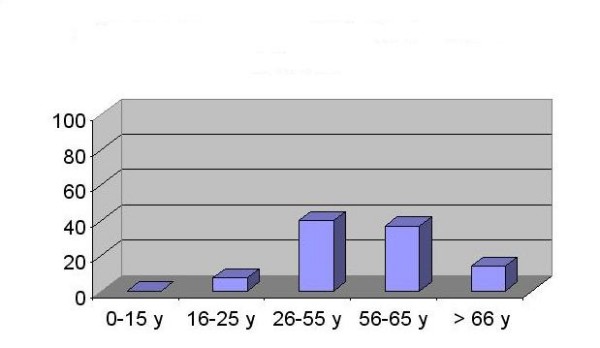
**Age distribution of prevalent regular HD patients**.

More than 80% of patients were between age 26 and 65 years old whereas only 7.5% of them were younger than 25 year, (Figure 2).

**Figure 3:**
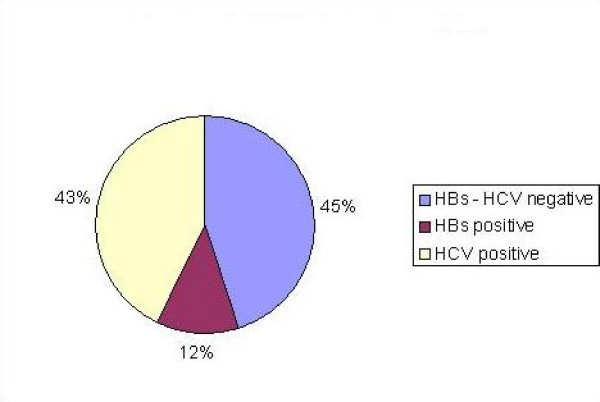
**HBV and HCV of prevalent regular patients HD patients**.

In this study, 70 patients (12%) were HBsAg positive, and 250 of them (43%) were anti HCV positive, (Figure 3).

## Disccusion

In patients on maintenance HD, the risk of hepatitis is still a serious problem despite the availability of serological tests and vaccines for hepatitis B virus infection and universal precaution standards and infection control measures. Available data suggest that HCV has become the most common cause of acute hepatitis in dialysis patients and dialysis staff members, following the implementation of infection control measures for HBV, [6].

In our study, the prevalence of HBsAg was 12%.

Results of the examination which were done with 165 HD patients from three units in Kosova (Prishtina, Prizren and Mitrovica) during first six months of 2003 showed that prevalence of anti HCV antibodies and HBsAg were 38.78%, respectively 24,24%. This study showed that duration of dialysis and number of transfusions are risk factors for development of HBV and HCV infection (p < 0, 05) [7].

The results of our last study demonstrate a decrease in prevalence of HBV positive HD patients during from 24, 24% to 12% over six years. This decrease of HBV infection in dialysis patients over the years despite implementation of universal precaution is a result of advent of recombinant human erythropoietin and HBV vaccination in last years. This prevalence is higher than in USA, Croatia, Japan, Casablanca, Iran, Jordan, Kenya, Saudi Arabia, Hong Kong and lower than India, Taiwan, Romania, Greece, Spain, Turkey and Brazil [8-23].

The HBV prevalence among the blood donors of Kosova is 4, 2%, which range Kosova to the second zone according to the CDC classification of the geographical expanding of the HBV infection. The HCV prevalence among the blood donors in Kosova is 0,3%, compared to other European countries this level of prevalence is relatively low [24].

Results of the study which were done with Kosovar refugees who had arrived in southern Italy in 1999 revealed that HBV infection seems to be at an intermediate level of endemicity. At the same time this study indicates that the level of endemicity of HCV infection in Kosovar population is low [25].

HCV prevalence in HD varies geographically, both within and between countries [26].

The reported anti-HCV seropositivity since 1999 ranges from low (1.9%) in the Slovenia [27] to high (80%) in Senegal [28]. HCV seroprevalence in the HD population was 59% in Bosnia and Herzegovina, 6.8% in Belgium, 16.3% in France, 6.1% in Germany, 10%–29% in Greece, 22.5%–32.1% in Italy, 75% in Moldavia, 3.4% in the Netherlands, 11% in Sweden, 7%–23.3% in the USA, 4% in the UK, 20.5% in Libya, 71% in Kuwait, 23.7% in Sudan, 19% – 41.7% in Tunisia, 8.4%–43.2% in Brazil, 6.7% in Mexico, 59.3% in Peru, 3.5% in Puerto Rico and 13.2% in Iran [29-47]

The prevalence of HCV among our HD patients is 43%, compared with above mentioned study [7] there is not a decrease in prevalence of HCV in our HD patients over last six years. In our opinion environment condition in our hemodialysis units are responsible for high prevalence of HCV in our HD patients.

## Conclusion

In summary, the prevalence of HBV and HCV in our HD patients is still high. These data emphasize the need for stricter adherence to infection control, barrier precaution and preventive behaviors with all patients.

## Competing interests

The authors declare that they have no competing interests.

## Authors' contributions

ST participated in the design of the study, comparing the results with other publications and drafted manuscript. HF participated in design of the study and performed the statistical analysis. YE carried about data collecting. TB participated in additional correction and design.

All authors have read and approved the final manuscript.
